# Comparative analysis of intestinal flora between rare wild red-crowned crane and white-naped crane

**DOI:** 10.3389/fmicb.2022.1007884

**Published:** 2022-12-01

**Authors:** Zhongsi Gao, Hongwei Song, Haiyan Dong, Xiaolong Ji, Zefeng Lei, Ye Tian, Yining Wu, Hongfei Zou

**Affiliations:** ^1^College of Wildlife and Protected Area, Northeast Forestry University, Harbin, China; ^2^Department of Genetics, College of Life Science, Northeast Forestry University, Harbin, China

**Keywords:** red-crowned crane, white-naped crane, intestinal microorganisms, high-throughput sequencing technology, Zhalong Nature Reserve

## Abstract

**Introduction:**

Animal intestines are extremely rich in microbial ecosystems. Numerous studies in different fields, such as epidemiology and histology, have revealed that gut microorganisms considerably mediate the survival and reproduction of animals. However, gut microbiology studies of homogeneously distributed wild cranes are still rare. This study aimed to understand the structural composition of the gut microbial community of wild cranes and elucidate the potential roles of the microorganisms.

**Methods:**

We used high-throughput sequencing to analyze the gut microbial community structure of wild cranes in the Zhalong Nature Reserve.

**Results:**

A total of 1,965,683 valid tags and 5248 OTUs were obtained from 32 fecal samples. Twenty-six bacteria phyla and 523 genera were annotated from the intestinal tract of the red-crowned crane. Twenty-five bacteria phyla and 625 genera were annotated from the intestine of the white-naped crane. Firmicutes, Proteobacteria, and Bacteroidetes are the dominant bacterial phyla in the intestinal tract of red-crowned cranes, while *Catellicoccus*, *Lactobacillus*, *Neisseria*, and *Streptococcus* were the dominant genera. The dominant bacterial phyla in the intestinal tract of white-naped cranes were Firmicutes, Proteobacteria, Bacteroidetes, Epsilonbacteraeota, Actinobacteria, and Fusobacteria. However, the dominant genera were *Catellicoccus*, *Lactobacillus*, *Neisseria*, *Campylobacter*, *Streptococcus*, *Anaerobiospirillum*, *Romboutsia*, *Turicibacter*, *Haemophilus*, and *Lautropia*. Firmicutes had significantly higher relative abundance in the intestine of the red-crowned than white-naped cranes (*P* < 0.05). However, the relative abundance of Actinobacteria and Bacteroidetes was significantly higher (*P* < 0.05) in the intestines of white-naped than red-crowned cranes. The diversity of the intestinal flora between the two crane species was significantly different (*P* < 0.05). Besides, the alpha diversity of the intestinal flora was higher for white-naped than red-crowned cranes. Eight of the 41 functional pathways differed in the gut of both crane species (*P* < 0.05).

**Discussion:**

Both species live in the same area and have similar feeding and behavioral characteristics. Therefore, host differences are possibly the main factors influencing the structural and functional differences in the composition of the gut microbial community. This study provides important reference data for constructing a crane gut microbial assessment system. The findings have implications for studying deeper relationships between crane gut microbes and genetics, nutrition, immunity, and disease.

## Introduction

The animal digestive tract is a complex and dynamic ecosystem with various microorganisms in the digestive tract (Hooper et al., 2001). Studies have shown that bacteria, archaea, and fungal microorganisms are the major groups of intestinal microorganisms (Muegge et al., 2011). Bacteria are the most common intestinal microorganisms (Chen et al., 2018) especially, the phyla that account for a large proportion of the total are Firmicutes, Bacteroidetes, and Proteobacteria (Sommer and Bäckhed, 2013). Numerous studies have proven that intestinal flora is crucial for host survival and reproduction (Hooper et al., 2001; McFall-Ngai et al., 2013). Intestinal flora controls energy homeostasis (Gabanyi et al., 2022), resistance disease and nutrition (Olvera-Rosales et al., 2021) and regulates the immune system (Grizotte-Lake et al., 2018).

Studies have shown that intestinal flora influences many physiological activities such as signaling, immune response, and nutrient metabolism in birds (Kohl, 2012; Grond et al., 2018). Bird gut flora can be influenced by various factors, such as habitat changes (Xie et al., 2016; Wu et al., 2018; Sun et al., 2019), weather changes (Wienemann et al., 2011; Zhang et al., 2020), host genotype (Trevelline et al., 2020; Gao et al., 2021), gender (Cui, 2019), age (Kohl et al., 2019), diet (Xie et al., 2016; Liu et al., 2020a), and health status (Ruiz-Rodríguez et al., 2009; Tong et al., 2018) among others. The dominant gut flora is key in maintaining the normal physiological functions of migratory birds (Grond et al., 2019). However, long-distance migratory birds may carry and transmit microorganisms across geographic areas (Canuti et al., 2019). Trevelline et al. (2019) and West et al. (2019) argue that understanding the microbial communities hosted by individual animals can improve conservation measures for rare wildlife and wild birds. Birds are important ecosystem indicator organisms, and the study of bird-gut microbes is ecologically important. Currently, there are approximately 10,000 bird species worldwide, nearly 2.5 times of mammals, yet research on bird gut microbes is only about 10% of the work on mammalian gut microbes (Grond et al., 2018).

Red-crowned (*Grus japonensis*) and white-naped (*Grus vipio*) cranes are large, wading, omnivorous birds. Adult cranes are monogamous and co-inhabit wetland environments. They are considered indicator species for wetland environmental quality (Zou and Wu, 2006; Wu, 2012). In February 2021, both crane species appeared in the National Class I Key Wildlife Protection of China’s National List of Key Wildlife Protection. Moreover, the IUCN Red List (2021) lists both the red-crowned crane and the white-naped crane as vulnerable (VU). The International Crane Foundation (ICF) estimates that 2,800–3,430 red-crowned cranes are in the wild globally. Furthermore, two major populations of red-crowned cranes exist worldwide: island resident populations and continental migratory populations. For white-naped cranes, the worldwide population in the wild is between 6,700–7,700 individuals. The western population winters in the middle and lower reaches of the Yangtze River in China, and the eastern population winters on the Korean Peninsula and Kyushu Island, Japan. Unfortunately, the populations of both crane species are declining in China.

Between March to November, wild red-crowned and white-naped cranes breed in some wetlands of Heilongjiang Province (Zou et al., 2018). The species of plants eaten by the cranes are similar during this breeding period, but the proportions vary. At courtship, the overlap of nutritional and ecological niches between the two populations was 91.67% (Wu, 2012). Although the two crane species are co-dominantly distributed, they differ in their selection and use of the microhabitats (Li, 2017).

Zhalong National Nature Reserve, located in the middle of the East Asia-Australasia bird migration corridor, has the largest reed wetland in Asia (Yang, 2015). The reserve acceded to the Convention on Wetlands of International Importance in 1992 as a waterfowl habitat. Zhalong National Nature Reserve focuses on protecting rare birds and wetland ecosystems, among them are birds including red-crowned and white-naped cranes, the oriental white stork (*Ciconia boyciana*), Eurasian Spoonbill (*Platalea leucorodia*), bustard (*Otis tarda*), and reed parrotbill (*Paradoxornis heudei*). The Zhalong Reserve is rich in crane resources and is an essential global crane research hotspot. Since the two crane species have similar breeding rhythms and reproductive patterns (Wu, 2012; Hao, 2013; Li, 2017), they can represent the interspecific differences in wild bird intestinal flora.

Therefore, this study used non-invasive sampling methods to collect feces from wild red-crowned and white-naped cranes of the same range. The 16S rRNA high-throughput sequencing technology was applied to analyze the composition and structure of crane intestinal flora. The study hypothesized that: (1) The gut microbial communities of cranes from the same habitats domain have similar composition. (2) The crane intestinal microbial communities perform similar functions. (3) The gut microbial communities of cranes from the same species are more similar than between individuals of different species.

## Materials and methods

### Ethics statement

Fourteen and eighteen fecal samples of wild red-crowned and white-naped cranes were collected from the natural environment using non-contact and non-invasive sampling methods without damaging cranes. The administration of Zhalong National Nature Reserve approved the sample collection.

### Sample collection

**FIGURE 1:**
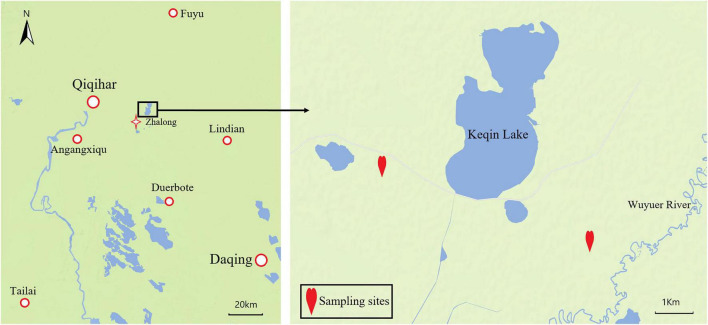
Map of geographical location of the Zhalong China and sampling point.

Test samples were collected from the Zhalong Nature Reserve (46°52′–47°32′N, 123°47′–124°37′E) between March and April 2021. The average annual precipitation and annual temperature in this area are 416 mm and 3.2^°^C, respectively. The climate during the sampling period is characterized by low precipitation, rapid warming, sandy winds, and partial dryness. Red-crowned cranes and white-naped cranes mainly eats maize (*Zea mays*), reeds (*Phragmites australis*), and *Suaeda glauca* in this period at Zhalong Nature Reserve (Wu, 2012). A preliminary survey revealed red-crowned, and white-naped cranes forage and rest in the farmland near Kachin Lake located within the reserve. Thus, that farmland was selected as the sampling site for this study (Figure 1).

To ensure proper species identification of each sample, we used telescopes to search for red-crowned crane and white-naped crane flocks, waited until the birds flew away and then collected fresh fecal samples. The interval distance for samples was more than 5 m to avoid individual repetition. The fresh and moist feces were kept in a portable liquid nitrogen tank and transported refrigerated to the lab as quickly as possible. The outside of each sample was cut and discarded to avoid contamination; the rest was stored at –20^°^C, transported to the laboratory, and stored at –80^°^C until further analysis for DNA extraction that could ensure the success and accuracy of subsequent sequencing (Supplementary Figure 1).

### Bacterial DNA extraction and sequencing

Total bacterial DNA was isolated from fecal samples using the MagPure Soil DNA LQ Kit (Producers: Magen, Cat. No.: D6356-02) following the manufacturer’s instructions. DNA integrity was measured using agarose gel electrophoresis. Next, PCR amplification of the V3-V4 hypervariable regions of the bacterial 16S rRNA gene was performed in a 25 μL reaction using universal primer pairs (343F: 5’-TACGGRAGGCAGCAG-3’; 798R: 5’-AGGGTATCTAATCCT-3’). The PCR products were purified with the Agencourt AMPure XP beads (Beckman Coulter Inc., CA, USA) and quantified using the Qubit dsDNA assay kit (Thermo Fisher Scientific, MA, USA). The library concentrations were adjusted accordingly, and libraries were sequenced on an Illumina NovaSeq6000 on two paired-end read cycles of 250 bases (Illumina Inc., CA, USA; OE Biotech Company; Shanghai, China). The raw data were submitted to the Sequence Read Archive at the NCBI database under BioProject accession number PRJNA835714.

### Bioinformatic analysis

Paired-end reads were assembled using the FLASH software (Reyon et al., 2012). Sequences with ambiguous and homologous sequences or with < 200 bp were discarded. The QIIME software (version 1.8.0) (Caporaso et al., 2010) retained reads where ≥ 75% of the bases scored Q20. Then, reads with chimeras were removed using VSEARCH (Rognes et al., 2016). Clean reads were subjected to primer sequences removal and clustering to generate operational taxonomic units (OTUs) using the VSEARCH software with a 97% similarity cutoff (Rognes et al., 2016). A representative read was selected from each OTU using the QIIME package. All representative reads were annotated and blasted against the Silva database (Version 132) using the RDP classifier (Wang et al., 2007).

### Statistical analysis

An alpha diversity analysis reflected the degree of bacterial diversity within the intestinal environment of the two crane species. The Chao1 (Chao, 1984), Shannon Wiener (Hill et al., 2003), Simpson (Simpson, 1949), and Good’s Coverage (Esty, 1986) indexes were calculated for the two types of crane intestinal flora based on uniform depth. The Wilcoxon sum rank test determined the difference in the alpha diversity indexes between the intestinal flora of the two crane species. Therefore, the number of actual observed OTUs (Observed Species) was used to plot alpha diversity dilution curves. Adonis analysis (i.e., PERMANOVA analysis) was used to test the difference between the different subgroups (*P* < 0.05 indicates a significant difference between groups, and the *R*^2^ value indicates the degree of the explained difference). A PCoA (Principal coordinates analysis) analysis based on a weighted Unifrac distance algorithm (considering the evolutionary relationships and species abundance) was used to compare the beta diversity between the gut of the red-crowned and white-naped cranes. Furthermore, the LEfSe (Linear discriminant analysis coupled with effect size measurements) analysis revealed the intestinal flora composition of the two crane species. All the statistical analyses were completed using the R package.

The functional prediction analysis of gut microbes was based on the 16S sequence data annotated on the Greengenes database (DeSantis et al., 2006). The PICRUSt (Phylogenetic investigation of communities by reconstruction of unobserved states) software predicted the gene functions of known microbial (Langille et al., 2013). The results were compared with the KEGG (Kyoto Encyclopedia of Genes and Genomes) database to find homologous genes and gene copy numbers. Thus, the corresponding KEGG Ortholog (KO) information was used for predicting functions. The abundance of each functional category was calculated based on the KO information and the corresponding OTU abundance. Finally, the Wilcoxon sum rank test established the functional differences between the gut microorganisms of the two crane species.

## Results

### Sequencing statistics

This study used the Illumina Novoseq platform to amplify and detect 16S rRNA sequences from the fecal microbiota of 14 red-crowned and 18 white-naped cranes. The 32 stool samples yielded 1,965,683 valid tags, including 43,917–69,817 valid tags per sample measuring 404.12–425.34 bp long. The sequences with ≥ 97% QC were classified as one OTU; thus, 5248 OTUs were obtained (651–2,130 OTUs in each sample). The observed OTUs of randomly selected sequences generated the rarefaction curves for the samples. Moreover, increasing the sequencing depth (the number of sampled sequences) increased the rarefaction curves of most samples, indicating a non-significant increase in new OTUs over the increasing sequencing depth (Supplementary Figure 2). The Good’s Coverage Index showed that the sequencing depth covered > 99% of the species in the sample, indicating that the experimental sampling was adequate.

### Comparison of the structure of the intestinal flora between the red-crowned and white-naped cranes

The red-crowned and the white-naped crane share 3088 OTUs, accounting for 58.84% of all the sampled OTUs. The red-crowned crane had 10.83% of the bacterial OTUs (375), and the white-naped crane had 57.80% of the endemic OTUs (1785) (Supplementary Figure 3). Simultaneously, we identified 72 OTUs as the core gut microbiota (the taxa) shared by all the individuals in each group of red-crowned and white-naped cranes (Supplementary Figure 4). The shared taxa were annotated to Firmicutes, Bacteroidetes, Proteobacteria, Actinobacteria, Fusobacteria, Epsilonbacteraeota, and Gemmatimonadetes.

**FIGURE 2:**
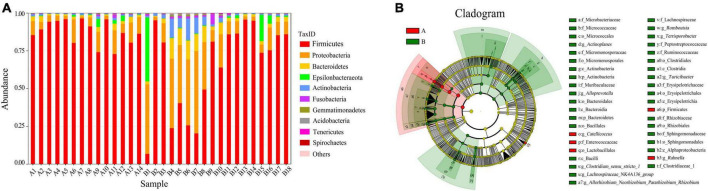
**(A)** Relative abundance bar plot of the relative abundance of the strains. Relative abundance (%) of the two cranes Top10 phyla. Red-crowned crane, A1–A14; white-naped crane, B1–B18. **(B)** Annotated branching diagram of differential strains. Red nodes indicate species with relatively high abundance in the red group, green nodes indicate species with relatively high abundance in the green group, yellow nodes indicate species with no significant difference in the comparison between the two groups, the node diameter size is proportional to the relative abundance size, each layer of nodes indicates the phylum/class/order/family/genus from the inside out. **(A)** Red-crowned crane, **(B)** white-naped crane.

Taxonomically, the red-crowned and white-naped cranes had 26 and 25 annotated intestinal phyla, 54 and 61 classes, 136 and 159 bacterial orders, 248 and 275 families, and 523 and 625 bacterial genera. We plotted the relative abundance bar plot of the intestinal flora of both cranes. The phylum-level flora in the intestine of red-crowned cranes consisted mainly of Firmicutes (87.55%), Proteobacteria (7.65%), and Bacteroidetes (2.14%). In contrast, the phylum-level flora in the intestine of white-naped cranes consisted mainly of Firmicutes (66.91%), Proteobacteria (18.83%), Bacteroidetes (4.80%), Epsilonbacteraeota (4.30%), Actinobacteria (3.30%), and Fusobacteria (1.01%) (Figure 2A). Besides, the genus-level flora in the intestine of red-crowned cranes consisted mainly of *Catellicoccus* (76.10%), *Lactobacillus* (7.87%), *Neisseria* (2.75%), and *Streptococcus* (1.06%). *Catellicoccus* (43.39%), *Lactobacillus* (11.05%), *Neisseria* (8.09%), *Campylobacter* (4.25%), *Streptococcus* (3.25%), *Anaerobiospirillum* (3.03%), *Romboutsia* (2.04%), *Turicibacter* (1.49%), *Haemophilus* (1.40%), and *Lautropia* (1.08%) dominated the genus-level flora in the intestine of the white-naped crane (Supplementary Figure 5).

The LEfSe analysis revealed the composition of the strains that differed between the groups and those that contributed more to the observed differences in each group (Figure 2B). In the gut of red-crowned cranes, Firmicutes (*P* < 0.05) were differentially dominant species at the phylum level, *Catellicoccus* (*P* < 0.01) and *Rahnella* (*P* < 0.01) at the genus level. However, the differentially dominant species in the gut of white-naped cranes were Actinobacteria (*P* < 0.05) and Bacteroidetes (*P* < 0.05) at the phylum level, *Clostridium_sensu_ stricto_1* (*P* < 0.05), *Romboutsia* (*P* < 0.001), *Lachnospiraceae_NK4A136_group* (*P* < 0.01), *Allorhizobium_Neorhizobium_Pararhizobium_Rhizobium* (*P* < 0.001), *Actinoplanes* (*P* < 0.05), *Terrisporobacter* (*P* < 0.05), *Alloprevotella* (*P* < 0.001), and *Turicibacter* (*P* < 0.01) at the genus level.

### Analysis of the diversity of the intestinal flora of two species of cranes

**FIGURE 3:**
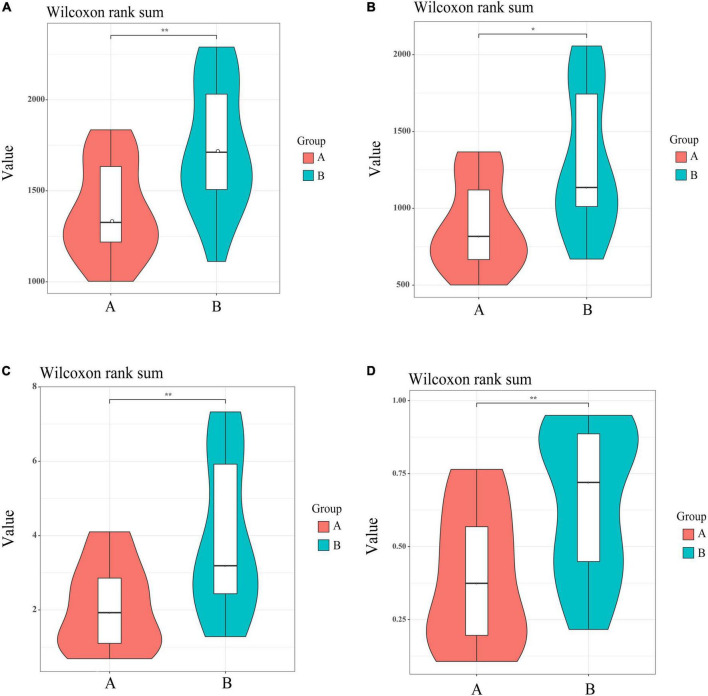
Alpha diversity index of microbial communities in violin map. **(A)** Representative chao1 Index, **(B)** representative observed species, **(C)** representative Shannon Index, **(D)** representative Simpson index. *Correlation significant at 0.05 level. **Correlation significant at 0.01 level.

**FIGURE 4:**
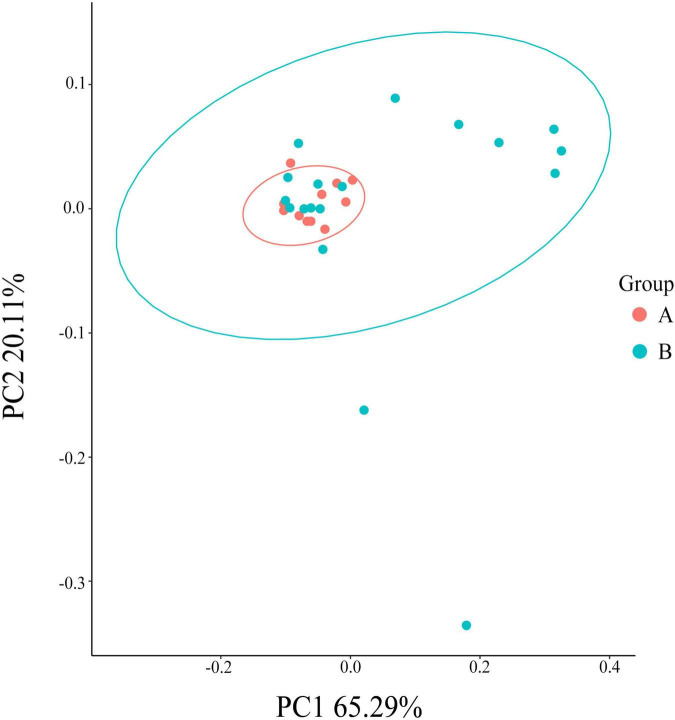
PCoA analysis graph of the colony. The same color in the graph is the same grouping. PC1 and PC2 are the first and second principal coordinates with the greatest explanation of the difference between samples.

The Chao index (*P* < 0.01), Shannon (*P* < 0.01), and Simpson indexes (*P* < 0.01) and observed Species (*P* < 0.05) showed that the gut flora of white-naped cranes was significantly more diverse than that of the red-crowned crane (Figure 3). Moreover, the Adonis analysis revealed significant differences between the intestinal flora of the two cranes species (*P* < 0.05, *R*^2^ = 0.139). PCoA analysis showed that individuals of the same species clustered together, indicating that both species had their own unique gut microbiota composition. Interestingly, most white-naped cranes were more dispersed, suggesting more specificity in the intestinal flora of white-naped cranes (Figure 4). The clustering of some white-naped cranes showed similarity to the red-crowned crane population, possibly due to the presence of both cranes foraging in the same area.

### Functional prediction analysis of the gut microbial community of the two crane species

We predicted functions of gut microorganisms in red-crowned and white-naped cranes by PICRUSt analysis and annotated 41 functional pathways at KEGG level2. The KEGG pathways for membrane transport (16.19%), carbohydrate metabolism (12.92%), replication and repair (8.62%), amino acid metabolism (8.09%), translation (5.77%), and poorly characterized (5.15%) were highly abundant in intestinal microorganisms from red-crowned cranes. In contrast, the gut microbes from white-naped cranes were highly abundant in KEGG pathways for membrane transport (14.15%), carbohydrate metabolism (11.36%), amino acid metabolism (9.10%), replication and repair (8.56%), translation (5.60%), energy metabolism (5.29%), and poorly characterized (5.11%).

**FIGURE 5:**
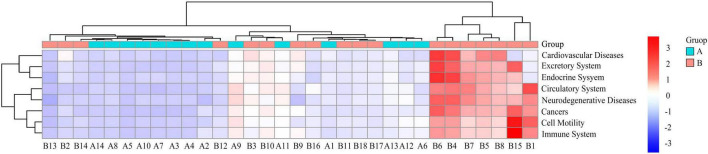
Heat map of differential functional clustering. Horizontal is the sample number; vertical is the functional annotation information; the clustering tree on the left side of the figure is the functional clustering tree; the upper clustering branches represent samples from different subgroups. The red color indicates a higher relative abundance of species, the blue color indicates a lower relative abundance of species. A, Red-crowned crane; B, white-naped crane.

Among the 41 functional pathways, cardiovascular diseases (*P* < 0.01) and cell motility (*P* < 0.01), excretory system (*P* < 0.05), immune system (*P* < 0.05), endocrine system (*P* < 0.05), cancers (*P* < 0.05), circulatory system (*P* < 0.05), and neurodegenerative diseases (*P* < 0.05) were significantly different between the two crane species (Figure 5). These eight differentially functional pathways were highly abundant in white-naped than red-crowned cranes.

## Discussion

The Zhalong Nature Reserve is among the most important breeding sites for wild and red-crowned cranes in China (Hao, 2013; Li, 2017). For the first time, we used high-throughput sequencing to analyze the intestinal flora of red-crowned and white-naped cranes in the wild. This study annotated 26 phyla and 523 genera in the gut of the red-crowned crane and 25 phyla and 625 genera in the gut of the white-naped cranes, which is significantly greater than previous results on the same species (Trevelline et al., 2020). This difference may be because the cranes in this study were wild and lived in a complex, variable environment. In contrast, the cranes in the Trevelline et al. (2020) study were caged and lived in a more homogeneous environment.

Firmicutes had the highest relative abundance in the gut of both crane species at the phylum level. This observation is consistent with that from the gut of 13 other cranes and many waterfowl species such as egrets (*Egretta garzetta*), black-crowned night heron (*Nycticorax nycticorax*), cormorant (*Phalacrocorax carbo*), and black swans (*Cygnus atratus*). Numerous studies have proven that Firmicutes are the most abundant in the intestines of almost all vertebrates, not only birds (Ding et al., 2017). This phenomenon may be greatly related to the function of Firmicutes, which degrade sugars and fats (Bäckhed et al., 2004) in intestinal food to produce energy and other nutrients for direct use by the host. Thus, they improve food utilization by the host (Flint et al., 2008). In addition, Firmicutes metabolize carbohydrates to produce short-chain fatty acids (SCFAs), which are also closely linked to host immune regulation (Atarashi et al., 2011; Zhang et al., 2015). Proteobacteria and Bacteroidetes were also common dominant groups in the intestines of both cranes, consistent with the dominant phylum in the intestines of chickens (*Gallus gallus domesticus*), turkeys (*Meleagris gallopavo*) (Wei et al., 2013), and 15 cranes species (Trevelline et al., 2020). The gut of swans also has similar dominant phyla composition (Wang et al., 2021). Proteobacteria are a very complex bacterial phylum that utilizes carbon-derived substances and is important in energy accumulation (Lu et al., 2012). Moreover, Proteobacteria have various physiological functions that are important for maintaining the homeostasis of their intestinal microbial communities and the healthy growth and development of their hosts (Tenaillon et al., 2010; Samanta et al., 2012). Bacteroidetes mainly ferment carbohydrates, polysaccharides, proteins, and bile acid, perform steroid metabolism, and promote fat accumulation (Salyers, 1984). This phylum produces butyric acid (Pryde et al., 2002), develops the host immune system, enhances host immunity (Hooper et al., 2001; Hooper, 2004), and are important for balancing the intestinal flora (Sears, 2005). In the spring, both red-crowned and white-naped cranes prefer foods with higher crude protein and crude fat and lower crude fiber (Wu, 2012). Moreover, accumulating Firmicutes, Proteobacteria, and Bacteroidetes benefits both cranes by obtaining sufficient energy and nutrients from these foods. These phyla also facilitate quick recovery of the copious energy consumed during the spring migration and prepare birds for the arrival of the breeding season. Genus *Catellicoccus* had the highest relative abundance in the gut of both crane species, consistent with the gut of the black-headed gull (*Chroicocephalus ridibundus*) and the whooper swan (*Cygnus cygnus*) in the Rongcheng area of Shandong Province (Wang et al., 2021). In contrast, the results differed from those of the cormorant, egret, and black-crowned night heron (Laviad-Shitrit et al., 2019). *Catellicoccus* is present in most waterfowl and can be used as indicator species for fecal contamination in water or environments contaminated by waterfowl feces (Koskey et al., 2014; Byappanahalli et al., 2015; Safaie et al., 2021). Besides, *Catellicoccus* also function in nutrient transport and bile acid hydrolysis (Góngora et al., 2021). *Lactobacillus* was highly abundant in the gut microbial communities of both cranes. Similarly, *Lactobacillus* was highly abundant in the intestines of other birds, including G. Canadensis (*Sandhill Crane*) (Trevelline et al., 2020), black-headed gulls (Laviad-Shitrit et al., 2019), black-necked cranes (*Grus nigricollis*) (Wang et al., 2020), and New Zealand parrots (*Strigops habroptilus*) (Waite et al., 2012). Moreover, *Lactobacillus* is a beneficial bacterium in the intestine as it synthesizes vitamins and amino acids required by the animal, promotes mineral absorption and regulates intestinal pH value. *Lactobacillus* inhibits the growth of pathogenic microorganisms, enhances the barrier function of intestinal epithelial cells, reduces inflammation, and enhances the body’s immune capacity (Artis, 2008; Milani et al., 2017).

In the intestinal flora of the two crane species, a LEfSe analysis revealed Firmicutes as the dominant phylum and *Catellicoccus* as the dominant genus. The differentially dominant phylum in the intestine of the white-naped crane were Actinobacteria and Bacteroidetes. Normally, the intestinal ratio of Firmicutes to Bacteroidetes (F/B) is related to the strength of fat metabolism in the animal body (Ley et al., 2005). In this study, the intestinal F/B in the red-crowned cranes was higher than that of the white-naped cranes, possibly because the red-crowned cranes have a larger body size compared to the white-naped cranes. Furthermore, the red-crowned cranes create larger nests and lay larger eggs during the breeding season (Hao, 2013; Li, 2017). All these differences normally require more energy consumption. Higher F/B also indicates that the cranes have higher fat metabolism and can obtain higher energy to sustain greater energy expenditure. Additionally, Actinobacteria are a dominant flora in the environment (Wink et al., 2017). The significant abundance of actinobacteria in the gut of the white-naped compared to the red-crowned crane may be due to the close association between the intestinal flora of the white-naped cranes and the environment.

The diversity analysis also showed that the alpha diversity of gut flora was significantly lower in red-crowned than white-naped cranes. Moreover, the Adonis and PCoA analyses showed significantly different structures of the intestinal flora of the two cranes (*P* < 0.05, *R*^2^ = 0.139). The intestinal flora diversity of black-necked cranes is significantly different between different regions (Wang et al., 2020), similar to the lesser white-fronted goose (*Anser erythropus*) (Liu et al., 2020a). During different migration seasons, the gut flora of hooded cranes (*Grus monacha*) was significantly different (Zhang et al., 2020). We selected wild red-crowned and white-naped cranes from the same distribution area in the spring. Thus, we excluded the influence of spatial and temporal factors on the intestinal flora diversity of the wild cranes. Studies on the lesser white-fronted goose have shown that food habits may influence intestinal flora diversity (Liu et al., 2020a). Wild red-crowned and white-naped cranes in the Zhalong Reserve feed on similar spring food types, and their trophic ecological niche overlap is approximately 90% (Wu, 2012). Therefore, food factors were not considered the main factors influencing the differences in the intestinal flora between the two crane species. Furthermore, Figure 4 showed that white-naped cranes appear to be separated into two clusters, one similar to red-crowned cranes and another separate cluster along the PC1. A possible explanation for this might be that the two species sharing the same foraging ground. According to our observation, white-naped cranes did not migrate to Zhalong wetland at the same time, some of that migrated at different times would forage in groups before the breeding season. Therefore, some individuals of the white-naped crane appear to be clustered and scattered. This result may be explained by the fact that individuals from different small migratory populations arrive at the Zhalong on different dates, while the intestinal flora of the white-naped cranes that arrived later may still retain the environmental adaptation characteristics of some migratory transit sites (Góngora et al., 2021).

By examining the intestinal flora of three species of geese, Yang et al. (2016) suggested that host species may influence the composition of intestinal flora. The gut microbial profile of 59 tropical bird species showed that host species had a greater effect on the gut flora than the host diet and inhabited region (Hird et al., 2015). Both results are consistent with our view that even with closely related species living in the same environment, the gut microbial community structure develops unique characteristics. The top five functional pathways annotated at level 2 contain functional genes that enrich membrane transport, carbohydrate metabolism, amino acid metabolism, and replication and repair. The results generally agree with the predicted gut microbial function of geese and red-crowned cranes in the middle and lower reaches of the Yangtze River (Yang et al., 2016; Dong et al., 2019). Moreover, the results are very similar to those of captive-bred chickens (Zhang, 2021). The predicted gut microbial functions of yak (*Bos mutus*) and donkey (*Equus asinus*) are also consistent with the current study (Liu et al., 2019). However, the predicted results of gut microbial functions of the Siberian tiger (*Panthera tigris altaica*) and tiger frog (*Hoplobatrachus rugulosus*) differed greatly from those of the crane (Liu et al., 2020b; Ning et al., 2020). Accordingly, we hypothesized that the closer the genetic distance, the more similar the gut microbial function in animals with similar food habits. Besides, the function of gut microbes is related to the adaptation of the host to the external environment (Newell and Douglas, 2014). For example, an enhanced corn (*Zea mays* L.) diet may promote the gut microbial metabolism of carbohydrates and lipids (Zheng et al., 2019).

In this study, wild red-crowned and white-naped cranes spent a lot of time foraging in the corn field. The high abundance of genes for gut microbial metabolic functions allows both cranes to efficiently obtain numerous nutrients from food to improve their adaptability to food and the environment. The abundance of the main functional groups of microorganisms in the gut of the two cranes was not significantly different (*P* > 0.05). However, the abundances of the dominant phylum and genera common to the gut of the two crane species were significantly different (*P* < 0.05). This difference suggests that the gut of different animals with the same domain distribution may be adapted to their respective habits by enriching different species of microorganisms with similar functions.

## Conclusion

This study analyzed the gut microbial communities of the red-crowned and white-naped cranes using 16S rRNA high-throughput sequencing. The results showed significant differences in the composition of gut microbial communities between the two crane species in the same range. However, the major functional groups were not significantly different. This study supports the view that host differences are the main reason for the different gut microbial communities. This data will help to develop more scientific conservation plans for cranes.

## Data availability statement

The datasets presented in this study can be found in online repositories. The names of the repository/repositories and accession number(s) can be found below: https://www.ncbi.nlm.nih.gov/, PRJNA835714.

## Ethics statement

This animal study was reviewed and approved by Animal Welfare and Animal Ethics Committee of Northeast Forestry University.

## Author contributions

ZG, YW, and HZ designed the experiments. ZG, HS, ZL, and YT completed the field sampling. HD, ZG, and XJ performed the data analysis and prepared the figures. ZG wrote the manuscript. YT and ZL contributed to the revision of manuscript. All authors contributed to the article and approved the submitted version.
