# Supplementary material for: Comparative analysis of intestinal flora between rare wild red-crowned crane and white-naped crane
Source: Front Microbiol. 2022 Dec 1;13:1007884. doi: 10.3389/fmicb.2022.1007884 (PMC9752901; doi:10.3389/fmicb.2022.1007884)
Supplement: Supplementary file 1 [file Data_Sheet_1.docx]

S1 Sample collection example diagram.

S2 OTU dilution curve. A1-A14 represent the samples of red-crowned crane stool, and B1-B18 represent the samples of white-naped crane stool.

S3 The Venn diagram. The Venn diagrams show the red-crowned crane (A) and white-napped crane (B) OTU of overlap.

S4 OTUs distribution petal diagram. The numbers in the Core represent the OTUs common to all samples, and the numbers on the petals represent the total OTUs of each sample minus the number of common OTUs.

S5. Cumulative histogram of the relative abundance of the strains. Relative abundance

(%) of the two crane species Top10 genera. Red-crowned crane: A1-A14; white-naped crane: B1-B18.
