# Supplementary figures and images for: Comparative analysis of intestinal flora between rare wild red-crowned crane and white-naped crane
Source: Front Microbiol. 2022 Dec 1;13:1007884. doi: 10.3389/fmicb.2022.1007884 (PMC9752901; doi:10.3389/fmicb.2022.1007884)

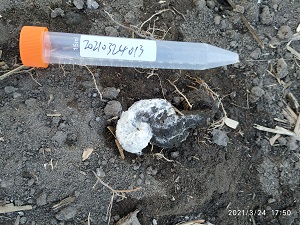

Supplement: Supplementary file 2 [file Image_1.JPEG]

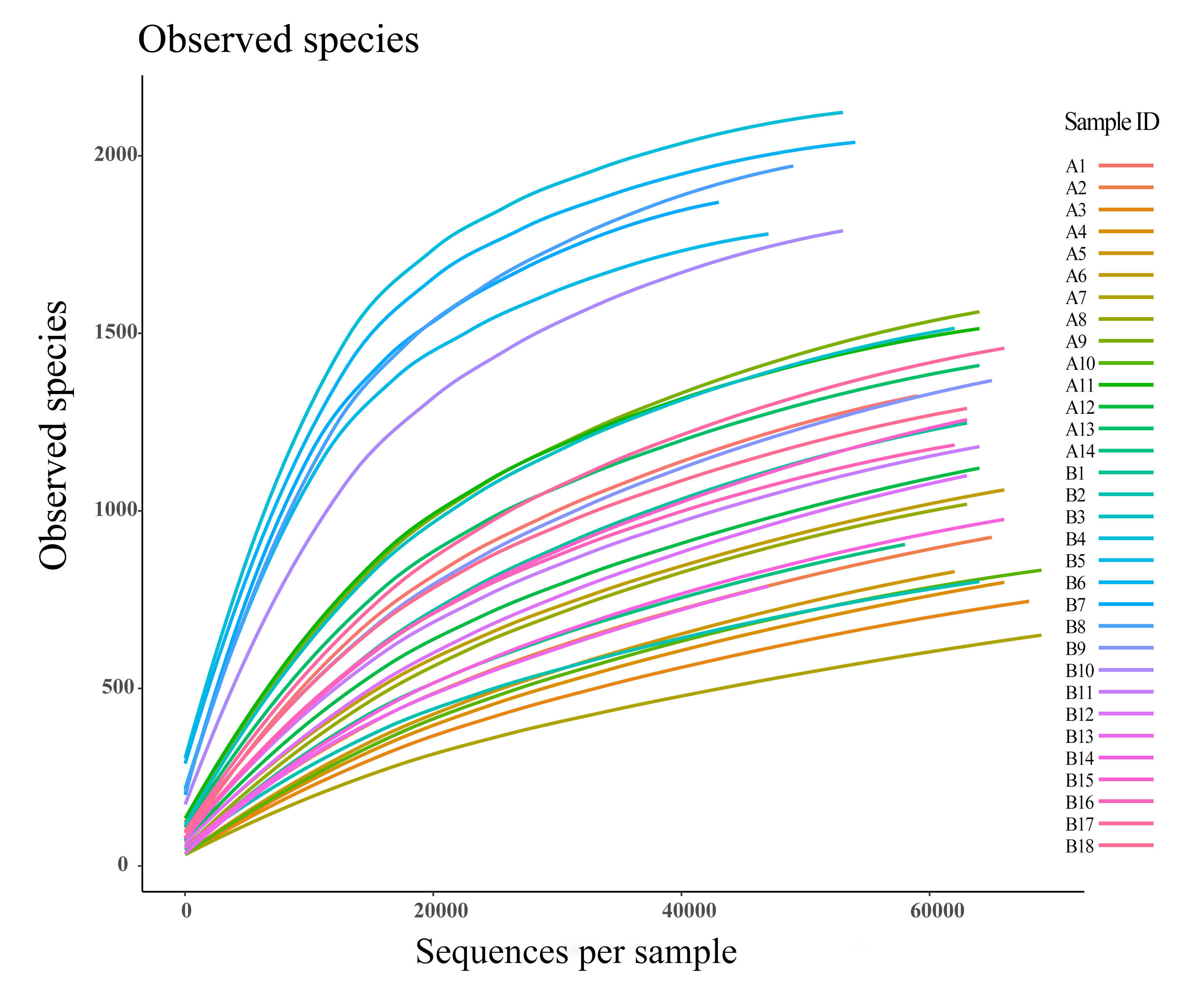

Supplement: Supplementary file 3 [file Image_2.JPEG]

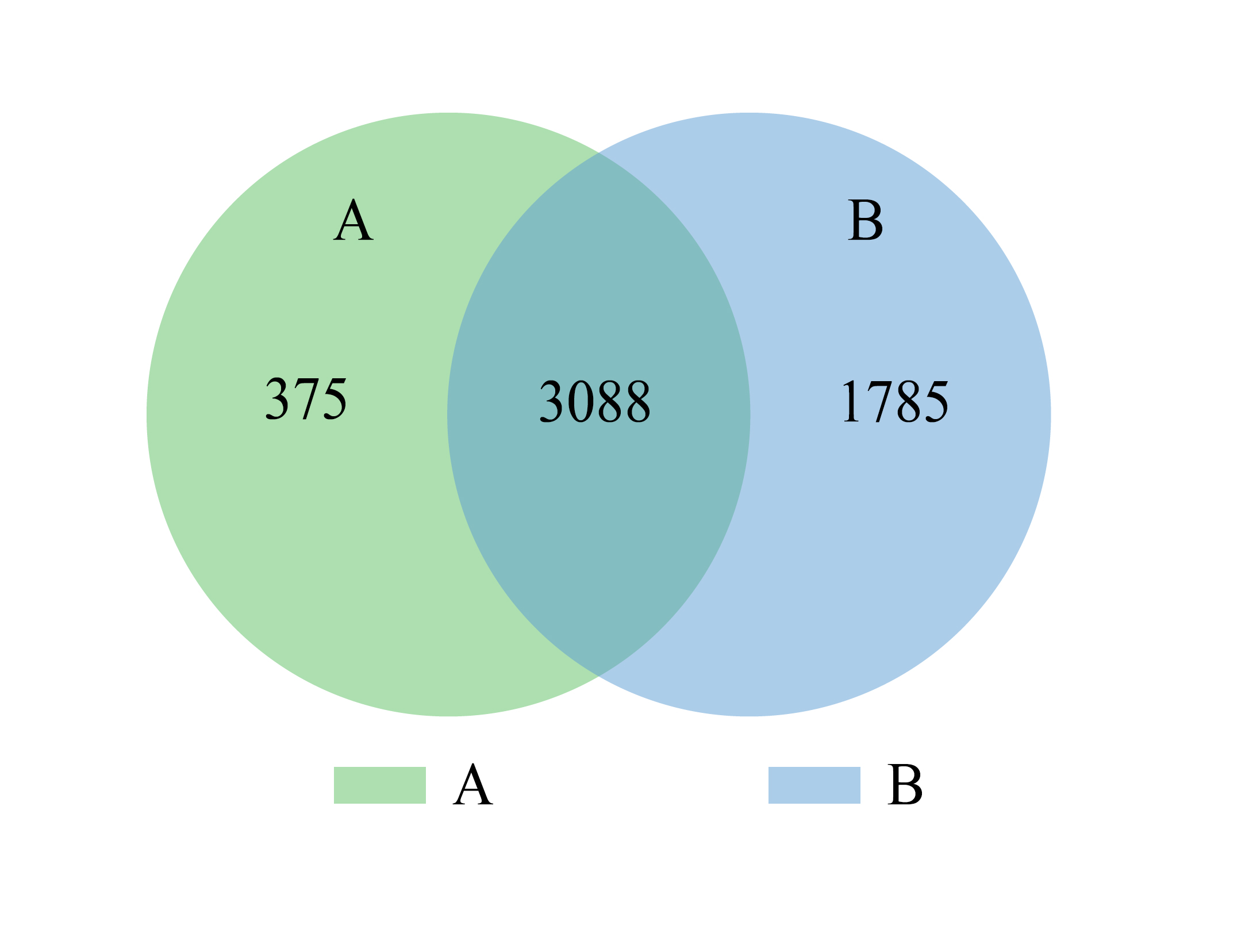

Supplement: Supplementary file 4 [file Image_3.JPEG]

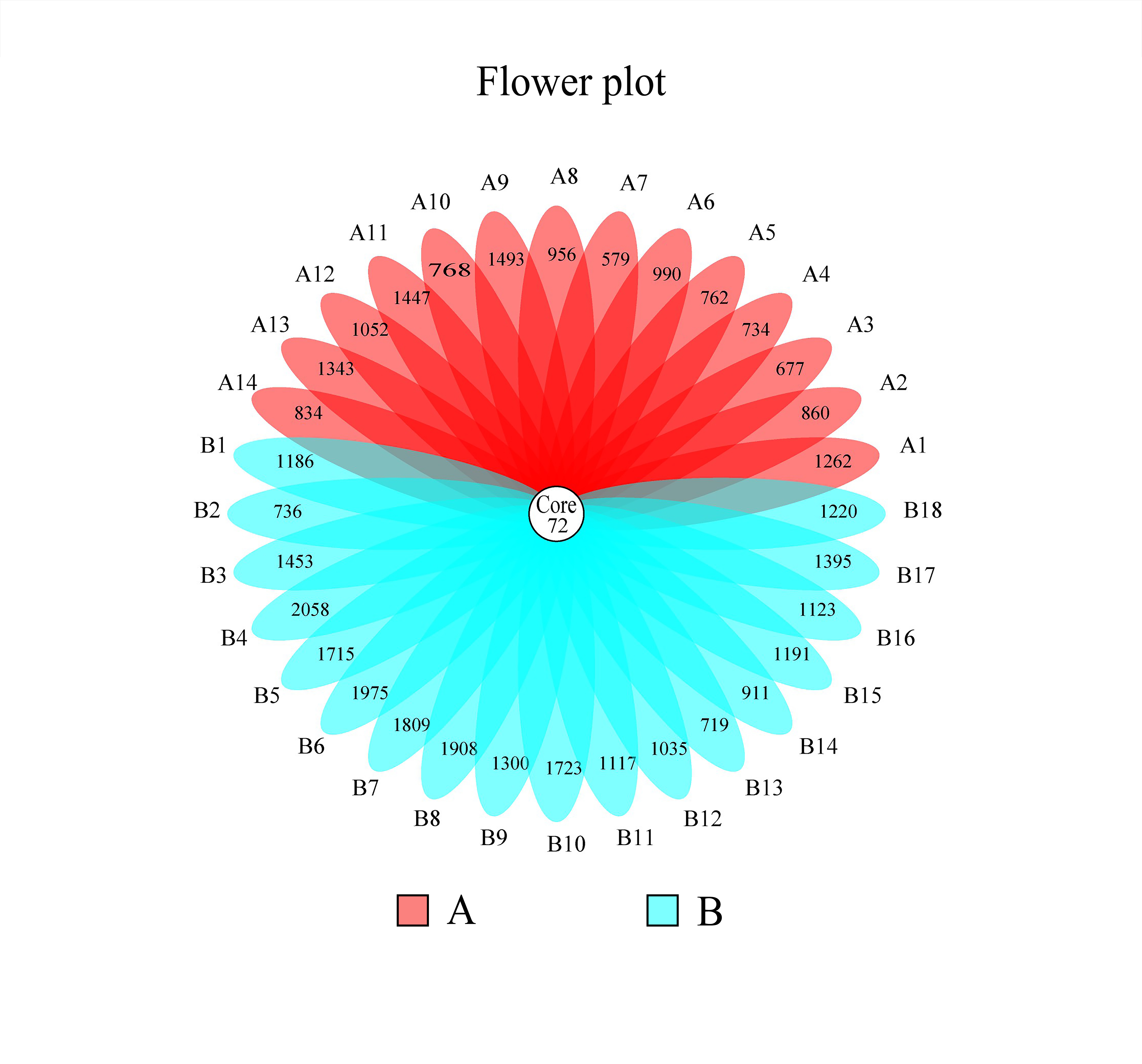

Supplement: Supplementary file 5 [file Image_4.JPEG]

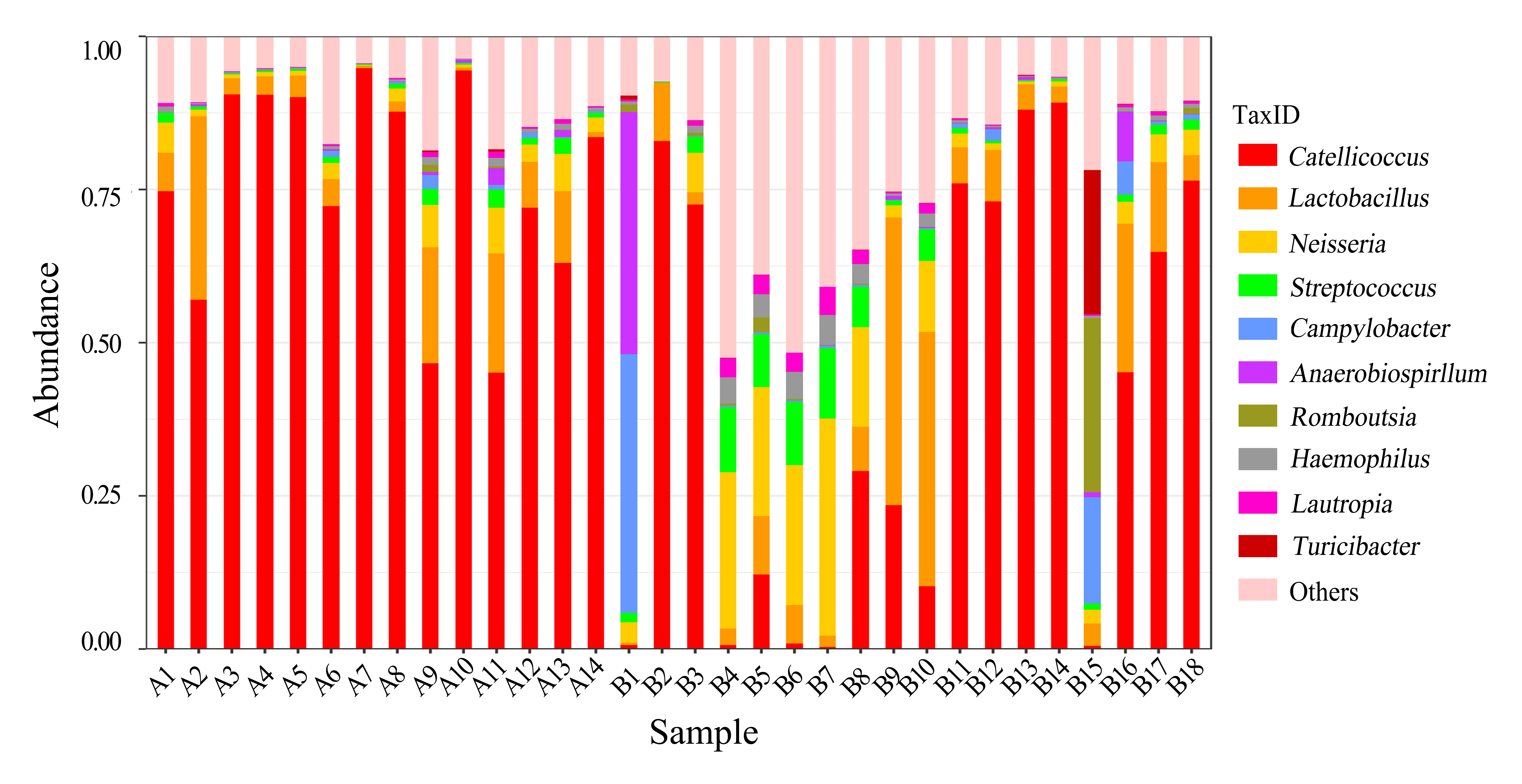

Supplement: Supplementary file 6 [file Image_5.JPEG]
